# Utilization of the Spanish Bisyllable Word Recognition Test to Assess Cochlear Implant Performance Trajectory

**DOI:** 10.3390/jcm14030774

**Published:** 2025-01-24

**Authors:** Meredith A. Holcomb, Erin Williams, Sandra Prentiss, Chrisanda M. Sanchez, Molly R. Smeal, Tina Stern, Amanda K. Tolen, Sandra Velandia, Jennifer Coto

**Affiliations:** 1 Department of Otolaryngology, University of Miami, Miami, FL 33136, USA; erin.williams@med.miami.edu (E.W.); svelandia@med.miami.edu (S.V.); jennifercoto@med.miami.edu (J.C.); 2 Section of Audiology, Cleveland Clinic, Cleveland, OH 44103, USA; 3 Department of Rehabilitative Services, Kapi’olani Medical Center for Women & Children, Honolulu, HI 96826, USA

**Keywords:** bisyllables, cochlear implant, cochlear implant outcomes, Spanish, Spanish-speaking outcomes, Spanish word recognition, Spanish cochlear implant test battery

## Abstract

**Objectives**: The aims of this study were to compare pre- and post-operative word recognition scores (WRSs) for the adult Spanish-speaking population and to describe their cochlear implant (CI) performance trajectory. **Methods**: A retrospective chart review (*n* = 115) was completed for Spanish-speaking post-lingually deafened adults who underwent a traditional CI evaluation and subsequent surgery between 2018 and 2023. Pre- and post-CI (3, 6, 12-month) Spanish Bisyllable WRSs and CI datalogging (hours per day) were collected for 66 subjects who met inclusion. Patients were, on average, 61.4 years of age (*SD* = 14.9) at the time of their first CI, and all were Hispanic and White (100%). **Results**: The outcomes of the 66 subjects who met the inclusion criteria were analyzed. Spanish Bisyllable WRSs improved at all post-CI test intervals, though the mean change between intervals showed a decreasing trend over time, with a plateau in WRSs occurring by 6 months post-CI. Time was a significant predictor of increased post-CI WRSs at 6 months (*p* = 0.004) and 12 months (*p* < 0.001). Sex, the implanted ear, electrode type, CI manufacturer, and datalogging hours did not significantly predict Bisyllable WRSs. **Conclusions**: This study used the largest cohort dataset to date to describe pre-and post-CI WRSs for Spanish-speaking adults. The post-CI performance trajectory is similar in Spanish-speaking CI recipients compared to English-speaking cohorts. This study is fundamental in providing evidence-based outcomes for Spanish-speaking CI recipients and will assist clinicians with pre-CI counseling based on realistic expectations.

## 1. Introduction

Understanding cochlear implant (CI) speech recognition outcomes for various patient populations is critical for pre-operative counseling and for mitigating barriers to accessing hearing healthcare treatment options. It is well known that a lack of timely CI referrals is a leading cause of the under-utilization of CI technology [1,2]. As such, multiple efforts over the last decade have been made to determine the optimal CI criteria and test battery for English-speaking adults in the United States (US), with the focus recently shifting from aided sentence to aided word scores as the primary indicator of CI candidacy [3,4,5,6,7,8]. This information is regularly used by hearing healthcare clinicians to identify potential English-speaking CI candidates during the CI evaluation and to classify CI recipients as poor or high performers post-CI [9]. Unfortunately, no universally adopted CI candidacy guidelines or recommended test batteries exist for Spanish-speaking adults in the US. To complicate matters further, there is known variability in the test materials, test presentation levels, and test conditions used to assess CI candidacy [10], particularly for the Spanish-speaking population [11]. 

With the increasing number of Spanish-speaking adults in the US [12] and the projected rise in the number of individuals with disabling hearing loss over the next several decades [13], it is essential to better understand the pre-CI evaluation and post-CI performance trajectory for this cohort of patients. Given that <3% of US audiologists speak Spanish [14], patients with a non-English language preference (NELP) [15] are at higher risk for inequitable access to hearing healthcare [16,17]. Additionally, Spanish-speaking adults often present to audiology clinics with variable unaided word recognition scores regardless of their degree of hearing loss [18], which can be confusing to clinicians who do not routinely work with this population and can lead to under-referral for CI technology. 

Furthermore, there is a paucity of research in the literature on CI outcomes for the US Spanish-speaking population. Velandia and colleagues [19] examined pre- and post-CI word and sentence scores for 24 Spanish-speaking CI recipients compared to 61 English-speaking CI recipients and for 12 bilingual CI recipients who served as their own controls. The mean CI word recognition score (WRS) was 65–72% across all post-CI test intervals using Spanish Bisyllable words [20,21] for both Spanish-speaking groups, and the mean WRS using English consonant-nucleus-consonant (CNC) [22] words was 55–57% for both English-speaking cohorts in this study. The most significant improvement in WRSs was observed in the first few months following CI activation for the English-speaking, Spanish-speaking, and bilingual groups [19], which is similar to what has been widely reported in the literature [23,24,25,26]. Given the comparable post-CI English and Spanish WRS performance trajectory, the Spanish Bisyllable word test was recommended for the adult Spanish CI test battery [19]. Interestingly, this suggestion aligns with CI guidelines from Spain [27,28] which state that a two-syllable Spanish word test is most appropriate for assessing CI candidacy in adults. 

The aim of this study was to compare pre- and post-CI Bisyllable WRSs at post-CI test intervals of 3, 6, and 12 months for a large cohort of CI recipients who identify Spanish as their primary language. The results of this study will provide a better understanding of the speech performance trajectory and expected outcomes for this population and will aid clinicians in providing Spanish-speaking patients with realistic CI expectations during pre-operative counseling. 

## 2. Materials and Methods

### 2.1. Study Design

A retrospective chart review was completed for 115 Spanish-speaking adults who were post-lingually deafened and underwent CI surgery for traditional candidacy at The University of Miami between 2018 and 2023. Of the 115 charts reviewed, 49 patients were excluded due to missing post-operative data and/or test results obtained with a monitored live voice. A total of 66 subjects with pre- and post-CI data were included in the study. 

Demographic information was collected, including sex, race, ethnicity, age at CI surgery, implanted ear, CI manufacturer, and electrode array. Bisyllable WRSs from pre-CI and post-CI test intervals of 3, 6, and 12 months were recorded. Only those with data for at least one post-operative test interval were included in the study. CI datalogging results were also collected for all post-CI test intervals. 

### 2.2. Participants

Roughly half the patients were female (*n* = 34; 51.5%), and all self-reported their ethnicity as Hispanic (100%) and White (100%). Patients were, on average, 61.4 years of age (*SD* = 14.9) at the time of their first CI surgery, and 51.5% were implanted in the left ear (*n* = 34). With respect to the CI manufacturer, 69.7% (*n* = 46) were implanted with a Cochlear [Sydney, Australia] CI, 16.7% (*n* = 11) with MED-EL [Innsbruck, Austria], and 13.6% (*n* = 9) with Advanced Bionics (AB) [Valencia, CA, USA]. More than half were implanted with a pre-curved electrode (*n* = 40; 60.6%). See Table 1 for demographics.

### 2.3. Procedures

Pre-operative CI evaluations were completed with the patient’s or clinic’s hearing aids verified to prescriptive targets with on-ear or test box measures. Testing for post-operative intervals was completed for the CI ear only, and the contralateral ear was plugged and muffed or masked if necessary. Aided word recognition testing was administered in a calibrated sound booth at a 60 dB SPL presentation level using the recorded Spanish Bisyllable word test [20,21] in quiet conditions. This test measure consisted of four lists of two-syllable paroxytone Spanish words recorded with a native Spanish speaker from Mexico. Each list has 50 words and starts with the carrier phrase “diga usted” (you say). The presentation level was confirmed via the sound level meter while words were played in the sound field. This step is necessary as a calibration noise track is not available on the Spanish Bisyllable test. Patients were positioned at 0 degrees azimuth for testing, and scores were recorded as the percentage of correctness. Pre-CI scores for the ear to be implanted were compared to post-CI scores for the CI ear. Datalogging for each test interval was also recorded.

### 2.4. Analysis

Statistical analyses were performed using SPSS (Version 28) and R (Version 2024.04.2). Data were analyzed to assess the relationships between predictor variables of interest (i.e., biological sex, age at implantation, implant manufacturer, and electrode type) and Spanish Bisyllable word recognition scores (WRSs) at 12 months post-activation. Standard diagnostic checks were performed to assess the assumptions of normality, linearity, and homoscedasticity. Transformations were applied where necessary to address any deviations from these assumptions. Simple linear regression was conducted to examine the relationship between timepoints (i.e., 3, 6, or 12 months post-CI) and WRSs. We also fit a linear mixed-effects model (LMM), with random intercepts included for participants in order to account for individual variability in baseline/pre-operative WRSs. The model was fit using the restricted maximum likelihood estimation. Fixed effects included all predictors of interest. Following LMM, an ANOVA was performed on the fitted model to evaluate the overall significance of each fixed effect. Post hoc comparisons were then conducted for any predictor that demonstrated an overall effect at an α < 0.05.

## 3. Results

### 3.1. Descriptive Statistics

**Figure 1:**
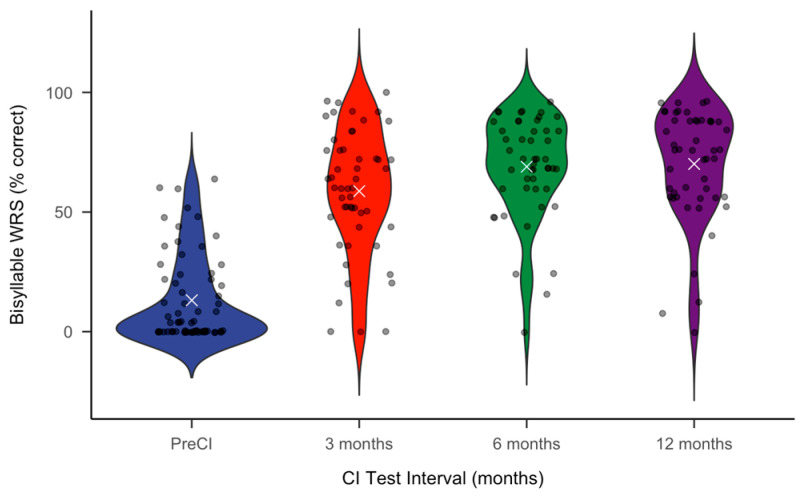
The violin plot for Bisyllable WRSs across test intervals. The violins illustrate the density of Bisyllable WRSs (% correct) at each timepoint; the white “×” marker is placed at the mean of the distribution for each timepoint.

Table 2 and Figure 1 summarize the WRS data. As shown in Table 2, average WRSs substantially increased from pre-CI to post-CI intervals. The largest increase occurred in the first 3 months post-CI, and smaller increases in WRSs were observed over time.

**Figure 2:**
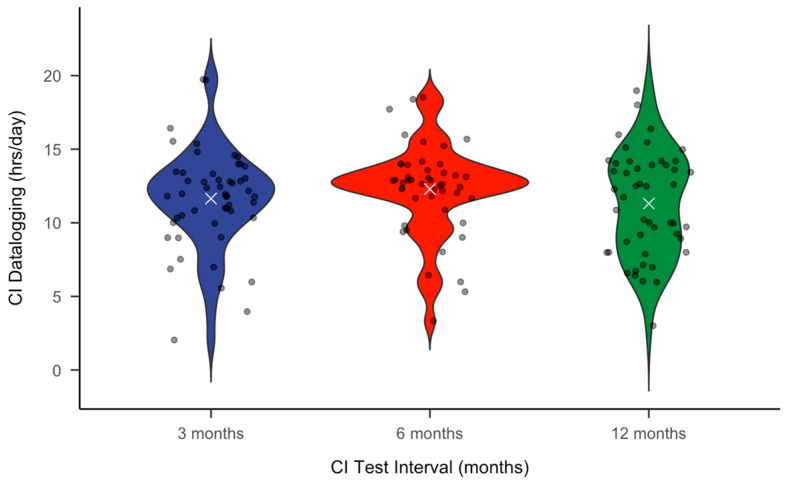
The violin plot for CI datalogging across test intervals. The violins illustrate the density of hours logged at each timepoint; the white “×” marker is placed at the mean of the distribution for each timepoint.

Table 2 and Figure 2 summarize the CI datalogging in hours per day. As shown in Table 2, the average time that the CI device was used is similar across post-CI intervals, with the largest quartile range occurring at 12 months post-CI.

### 3.2. Simple Linear Regression

Following simple linear regression, we observed a significant positive relationship between timepoint and log-transformed WRSs, with the following regression equation: log(*WRS Score*) = 1.21 + 0.86·*Timepoint*, where WRSs increased by an average of 0.86 units on the log scale per timepoint across all test intervals (pre-CI, 3 mo, 6 mo, 12 mo). This model accounted for 37% of the variance in WRSs (R^2^ = 0.370, F(1, 215) = 126.5, *p* < 0.001), suggesting a moderate association between timepoint and score improvement. Figure 1 summarizes the distribution of the WRS performance using a box and whisker plot.

### 3.3. Linear Mixed Effects Models

Linear mixed-effects models with patients as random intercepts were generated to examine the effects of time, biological sex, implant sidedness (i.e., left vs. right), the implant manufacturer, electrode type, and CI datalogging on Spanish Bisyllable WRSs. Estimated marginal means (EMMs) and pairwise comparisons were calculated using the emmeans package, with Bonferroni corrections applied to account for multiple comparisons. Following LMM (Table 3), time was found to be a significant predictor of increased WRSs, both at 6 months (β = 7.27, SE = 2.46, t(77) = 2.96, *p* = 0.004) and at 12 months (β = 10.47, SE = 2.51, t(77) = 4.17, *p* < 0.001) compared to pre-operative values. Males had lower scores than females (β = −9.70, SE = 5.06, t(60) = −1.92, *p* = 0.060), which approached statistical significance. Those with right-sided implants demonstrated higher WRSs compared to left-sided implant recipients (β = 9.50, SE = 5.25, t(60) = 1.81, *p* = 0.075), which also approached significance.

Additionally, Cochlear devices were found to be associated with higher WRSs compared to AB implants (β = 12.54, SE = 7.58, t(60) = 1.65, *p* > 0.05), and MED-EL [Innsbruck, Austria] implants were not significantly different from AB (β = −4.25, SE = 10.20, t(60) = −0.42, *p* = 0.678). The electrode type did not significantly affect WRSs (β = −0.32, SE = 6.27, t(60) = −0.05, *p* = 0.960), and datalogging hours also showed no significant association with Spanish Bisyllable WRSs (β = 0.11, SE = 0.60, t(77) = 0.18, *p* = 0.860). 

Next, ANOVA was conducted to evaluate the overall effect of the significant categorical predictors of the implant location (F(1,60) = 5.25, *p* = 0.03) and implant manufacturer (F(2,60) = 3.93, *p* = 0.03). Participants receiving left-sided implants had an average post-CI WRS of M = 54.9 (SE = 4.19, 95% CI: [46.5, 63.3]), whereas those with right-sided implants scored M = 64.4 (SE = 4.29, 95% CI: [55.8, 73.0]). Post hoc pairwise comparisons revealed that, on average, patients with right-sided implants had higher WRSs than those with left-sided implants (ΔM = −9.5, SE = 5.25, t(60) = −1.81, *p* = 0.075), though this did not reach statistical significance. Additionally, pairwise comparisons among the manufacturers revealed no significant differences following the Bonferroni correction. AB implant recipients scored M = 56.9 (SE = 6.83, 95% CI: [43.2, 70.6]), Cochlear recipients scored M = 69.4 (SE = 3.45, 95% CI: [62.5, 76.3]), and MED-EL implant recipients scored M = 52.6 (SE = 7.45, 95% CI: [37.7, 67.5]). Though Cochlear CI recipients had higher scores than AB implants, the difference was not statistically significant (ΔM = −12.54, SE = 7.58, t(60) = −1.65, *p* = 0.31). No significant differences were observed between AB and MED-EL implants (ΔM = 4.25, SE = 10.20, t(60) = 0.42, *p* = 1.00) or between Cochlear and MED-EL recipients (ΔM = 16.79, SE = 8.97, t(60) = 1.87, *p* = 0.20).

## 4. Discussion

Hispanic individuals in the US with NELP are at disproportional risk for healthcare disparities [15,16,17], including hearing healthcare [11,29]. It has been recommended that this population should be referred for a formal CI evaluation if the ear to be implanted has a pure-tone average (PTA) worse than 60 dB HL [11,18]. However, the CI evaluation and post-operative test batteries are not well defined, which inevitably leads to under-referral for Spanish-speaking CI candidates [30]. Additionally, the vast majority of US audiologists are monolingual English speakers [14] who report that they are unfamiliar and uncomfortable with how to manage Spanish-speaking patients with hearing loss [31,32].

Our study reports pre- and post-CI WRSs for the largest cohort (*n* = 66) of Spanish-speaking US adults found in the literature. Our findings revealed that aided Spanish Bisyllable WRSs improved from 13% pre-CI to 59% at 3 months post-CI, 69% at 6 months post-CI and 70% at 12 months post-CI. These results are in good agreement with Velandia et al. [19], who reported mean Spanish Bisyllable WRSs of 65% across all post-operative test intervals for 24 months post-CI. On average, Spanish-speaking CI recipients score slightly better than their English-speaking counterparts. According to a recent meta-analysis by Ma et al. [23], English-speaking patients have a group post-CI mean WRS of 60%. This difference in performance is not surprising as Spanish-speaking adults often score better on WRSs regardless of the degree of hearing loss, [18] which is likely due to the inherent phonemic and syntactic differences between the two languages [33,34,35,36]. 

It is well documented that English-speaking CI recipients typically experience significant improvements in WRSs by 3–6 months post-CI and plateaus in speech outcomes thereafter [9,23,24,25]. Likewise, our study found that the largest initial improvement in Bisyllable WRSs is from pre-CI to 3-month post-CI, with continued minimal increases documented until the 12-month test interval. This corroborates the results from Velandia et al. [19]. Additionally, our study revealed an overall WRS improvement of 57% from pre-CI to 12 months post-CI, which is comparable to the 51% change noted by Ma and colleagues [23] for the same time period in English-speaking patients. Thus, while our finding is that post-CI Spanish Bisyllable WRSs are higher than what is typically reported for English-speaking adults, the overall mean WRS improvement from pre- to post-CI is similar between the two groups.

It should be noted that when examining individual performance instead of group means (Figure 1), wide variability in performance was observed, which is also comparable to the English-speaking CI population [4,5,6,9,23,24,25,37]. Multiple efforts in predicting WRS outcomes for English-speaking CI recipients have yielded little concrete results to date [9,36,37,38,39,40,41,42,43,44,45]. Even so, we examined whether sex, the implanted ear, CI manufacturer, electrode array, or hours of device use were associated with improved WRSs for the Spanish-speaking population. On average, patients with right-ear CIs scored approximately 10% higher on Spanish Bisyllables than those with left-ear implants. Similarly, females had 10% better WRSs than males post-CI. While both sex and the CI ear were found to approach significance, they ultimately were not statistically significant. 

Cochlear CI recipients were associated with better post-operative performance (70% WRS) compared to those with AB (57%) or MED-EL (52%). This difference is not significant, but it is worth noting and should be investigated further. For the southeast US region, Cochlear has the longest history of all US CI companies offering language-appropriate resources for Spanish-speaking CI recipients, including a bilingual clinical specialist, a bilingual consumer specialist, and bilingual recipient services. A lack of culturally appropriate counseling and patient education are recognized as barriers for patients who are Hispanic with NELP [15,16,29,46]. Therefore, the availability and utilization of Cochlear’s Spanish resources may have contributed to patient adherence to post-CI clinical recommendations (device use, aural rehab), which, in turn, yielded better outcomes [29,46,47,48]. Our sample size for Cochlear was larger than other manufacturers, which could have also played a role in our results. Regardless of the CI company, the electrode type (lateral wall vs. pre-curved) did not significantly affect WRSs for this population. 

CI datalogging also showed no significant association with post-CI Spanish Bisyllable WRSs, which contrasts with findings for English CNC sores [49,50,51,52]. Interestingly, we found that male and female Spanish-speaking CI recipients, on average, wear their CI devices greater than 11 h per day at each post-CI test interval. Recent recommendations for English-speaking CI recipients state that patients should wear the device at least 11.5 h per day to reach their optimal CNC WRS by 3 months post-CI [49]. Similar studies have yet to be completed for Spanish-speaking adults. However, clinicians should consider the same datalogging recommendation for Spanish-speaking CI users for now. As shown in Figure 2, there is some individual variability in CI datalogging for our Spanish-speaking patients, similar to what has been published for English-speaking adults [49,50,51,52]. Interestingly, we found more individual datalogging variability at 12 months post-CI than for the other test intervals. While we do not completely understand this, it is possible that because patients plateaued in WRSs by 6 months, they became more relaxed with adhering to datalogging recommendations. This should be investigated further in future studies. Overall, our datalogging findings may be influenced by the fact that we routinely offer pre- and post-CI counseling via the use of interpreters and language-appropriate written recommendations and realistic expectations. We are also fortunate to have several bilingual audiologists on our CI team, which is critical as most CI recipients report that audiologists are the primary source of CI information [53].

There are several limitations to this study. First, the study is retrospective in design, and only certain parameters were included in the data collection. Future studies should consider the inclusion of sentence test scores, the use of patient-reported outcome measures to assess quality of life improvements, and/or the Cochlear Implant International Classification of Functioning, Disability, and Health Model (CI–ICF) [54] to better report on holistic outcomes for this population post-CI. Second, our study reports on Spanish-speaking adults implanted in only one region of the US. A multi-center study with patients from a wide variety of cities and regions in the US and other countries could be considered at a later time. 

In summary, our results support the use of commercially available Auditec Spanish Bisyllable word lists [21] for the pre- and post-CI test battery as its performance trajectory mimics that of the English CNC word test. While post-CI WRSs may be slightly better for the Spanish-speaking population, the mean improvement from pre- to post-CI is comparable to the English-speaking population. As such, Spanish-speaking adults who score <50% for Spanish Bisyllable pre-CI-aided WRSs should be considered for CI candidacy, similar to the recommendation for English-speaking CI candidates [3,4,5,6]. 

## 5. Conclusions

Post-CI word recognition performance trajectory and outcomes are similar for the Spanish-speaking population compared to what has been widely reported in the literature for the English-speaking population. The results of this study provide clinicians with evidence-based information to assist with counseling Spanish-speaking CI candidates and recipients on realistic expectations relative to post-CI outcomes. 

## Figures and Tables

**Table 1:** Participant characteristics.

Characteristic	*n* (%)	Mean (*SD*)
Total CIs	66	
Age at CI Surgery (yrs)		61.4 (14.9)
Range		21–90
Sex		
Female	34 (51.5)	
Male	32 (48.5)	
Ethnicity		
Hispanic	66 (100)	
Race		
White	66 (100)	
Ear Implanted		
Left	34 (51.5)	
Right	32 (48.5)	
Pre-CI PTA (dB HL)		
Implanted Ear		94.6 (15.9)
Non-CI Ear		85.9 (20.8)
Unaided Pre-CI WRS (%)		
Implanted Ear		14.67 (18.2)
Non-CI Ear		33.9 (30.4)
CI Manufacturer		
Advanced Bionics	9 (13.6)	
Cochlear	46 (69.7)	
MED-EL	11 (16.7)	
Electrode Type		
Straight	36 (39.4)	
Pre-Curved	40 (60.6)	

Abbreviation: CI = cochlear implant; PTA = pure-tone average of 0.5, 1, 2, kHz; WRS = word recognition score.

**Table 2:** Single means for Bisyllable WRS and datalogging at each timepoint.

Test Interval	Biysllable Word Recognition Score				CI Datalogging Hours per Day			
	Mean (%)	*SD*	Range	*n*	Mean (hr)	*SD*	Range	*n*
Pre-op	13.12	18.20	0–64	66	--	--	--	--
3 Months	58.73	25.95	0–100	52	11.64	3.35	2–19.7	51
6 Months	68.94	21.26	0–96	49	12.29	3.08	3–18.5	47
12 Months	70.08	23.28	0–96	50	11.31	3.54	3–19	48

CI = cochlear implant; *n* = sample size; *SD* = standard deviation.

**Table 3:** Linear mixed-effects models examining the effects of time, biological sex, the implanted ear, implant manufacturer, electrode type, and device hours logged on Spanish Bisyllable WRSs with patients as random intercepts.

Effect	Value	SE	df	t-Value	*p*-Value
(Intercept)	49.98	9.60	77	5.21	<0.001 ***
Timepoint (6 months)	7.27	2.46	77	2.96	0.004 **
Timepoint (12 months)	10.47	2.51	77	4.17	<0.001 ***
Sex (Male)	−9.70	5.06	60	−1.92	0.060
Ear Implant (Right)	9.50	5.25	60	1.81	0.075
Manufacturer (Cochlear)	12.54	7.58	60	1.65	0.103
Manufacturer (MED-EL)	−4.25	10.20	60	−0.42	0.678
Electrode (Straight)	−0.32	6.27	60	−0.05	0.960
Datalogging	0.11	0.60	77	0.18	0.860

Abbreviations: SE = standard error; df = degrees of freedom. Significance values are denoted as follows: ** = *p* < 0.01; *** = *p* < 0.001.

## Data Availability

Data supporting the reported results can be found by contacting the corresponding author.

## Abbreviations

CI	Cochlear implant
US	United States
WRS	Word recognition score
NELP	Non-English language preference
AB	Advanced Bionics
SD	Standard deviation
CNC	Consonant nucleus consonant
LMM	Linear mixed-effects model
PTA	Pure-tone average
EMM	Estimated marginal mean
